# High-efficiency broadband second harmonic generation in single hexagonal GaAs nanowire

**DOI:** 10.1038/s41598-017-02199-w

**Published:** 2017-05-19

**Authors:** Jing Wang, Ying Yu, Yu-Ming Wei, Shun-Fa Liu, Juntao Li, Zhang-Kai Zhou, Zhi-Chuan Niu, Si-Yuan Yu, Xue-Hua Wang

**Affiliations:** 1 State Key Laboratory of Optoelectronic Materials and Technologies, School of Physics, Sun Yat-Sen University, Guangzhou, 510275 China; 2 State Key Laboratory of Optoelectronic Materials and Technologies, School of Electronics and Information Technology, Sun Yat-Sen University, Guangzhou, 510275 China; 3 State Key Laboratory of Superlattices and Microstructures Institute of Semiconductors, Chinese Academy of Sciences, Beijing, 100083 China; 4 Photonics Group, Merchant Venturers School of Engineering, University of Bristol, Bristol, BS8 1UB UK

## Abstract

In this paper, we investigate second harmonic generation in a single hexagonal GaAs nanowire. An excellent frequency converter based on this nanowire excited using a femtosecond laser is demonstrated to operate over a range from 730 nm to 1960 nm, which is wider than previously reported ranges for nanowires in the literature. The converter always operates with a high conversion efficiency of ~10^−5^ W^−1^ which is ~10^3^ times higher than that obtained from the surface of bulk GaAs. This nanoscale nolinear optical converter that simultaneously owns high efficiency and broad bandwidth may open a new way for application in imaging, bio-sensing and on-chip all-optical signal processing operations.

## Introduction

Second harmonic generation (SHG) is a second-order nonlinear optical process in which an optical wave with frequency *ω* is converted into a second wave at a doubled frequency of 2*ω*. SHG is well known to be a forbidden process in materials that have a center of inversion symmetry, but it can occur in noncentrosymmetric materials or at the surface of a material with any symmetry group^1^. As the first nonlinear optical effect to be found after the invention of the laser, SHG was first demonstrated in crystalline quartz by Franken *et al*. in 1961^2^. Since then, SHG has been a subject of intense theoretical and experimental study in many optical materials, including potassium dihydrogen phosphate crystals^3^, nematic liquid crystals^4^, GaP photonic crystal waveguides^5^, magnetic metamaterials^6, 7^, left-handed metamaterials^8^, AlGaAs microdisks^9^, quantum dots^10^, and biological tissues^11^.

Recently, SHG in nanowires (NWs), which are one-dimensional nanostructures with a thickness or diameter of tens of nanometres or less and unconstrained length, has attracted increasing attention because it has potential for use in a wide variety of applications, including generation of nanoscale coherent light sources^12^, optical correlator^13^, integrated nanophotonic components^14^, highly localised excitation^15^, crystal structure identification^16, 17^, and nonlinear optical microscopy^18–20^. As previously reported in the literature, NWs composed of several different materials, including alkaline niobates^21^, ZnO^22–24^, ZnTe^20^, ZnSe^25^, ZnS^16^, CdS^13, 26^, GaAs^17, 19, 27^, GaN^18^, GaP^28, 29^, InP^30^, perovskite Na_0.5_Bi_0.5_TiO_3_^31^, graphene^32^ and Pt^33^, can be excited to generate second harmonic light. Among these materials, GaAs NWs are excellent candidate materials for SHG because of the high second-order nonlinear optical susceptibility of GaAs.

In 2013 He *et al*. demonstrated that a vertical free-standing GaAs-NW array could generate broadband SHG signals when excited using femtosecond (fs) lasers and briefly mentioned that an isolated single NW is also capable of producing broadband SHG signals^34^. However, the conversion efficiency of the broadband SHG signals that were generated, which is very important for SHG in practice^24, 26^ was not given. In this work, we systematically investigated SHG using a single hexagonal GaAs NW. We found that a single GaAs NW can be excited using a fs laser to generate second harmonic light over a broad wavelength range from 730 to 1960 nm, which is wider than the ranges reported for NWs in the literature such as that of ref. 34. Additionally, the conversion efficiency of the generated broadband SHG signals is insensitive to the excitation wavelength and can reach the order of ~10^−5^ W^−1^, which is three orders of magnitude higher than that which occurs at the surface of bulk GaAs.

## Results and Discussion

**Figure 1:**
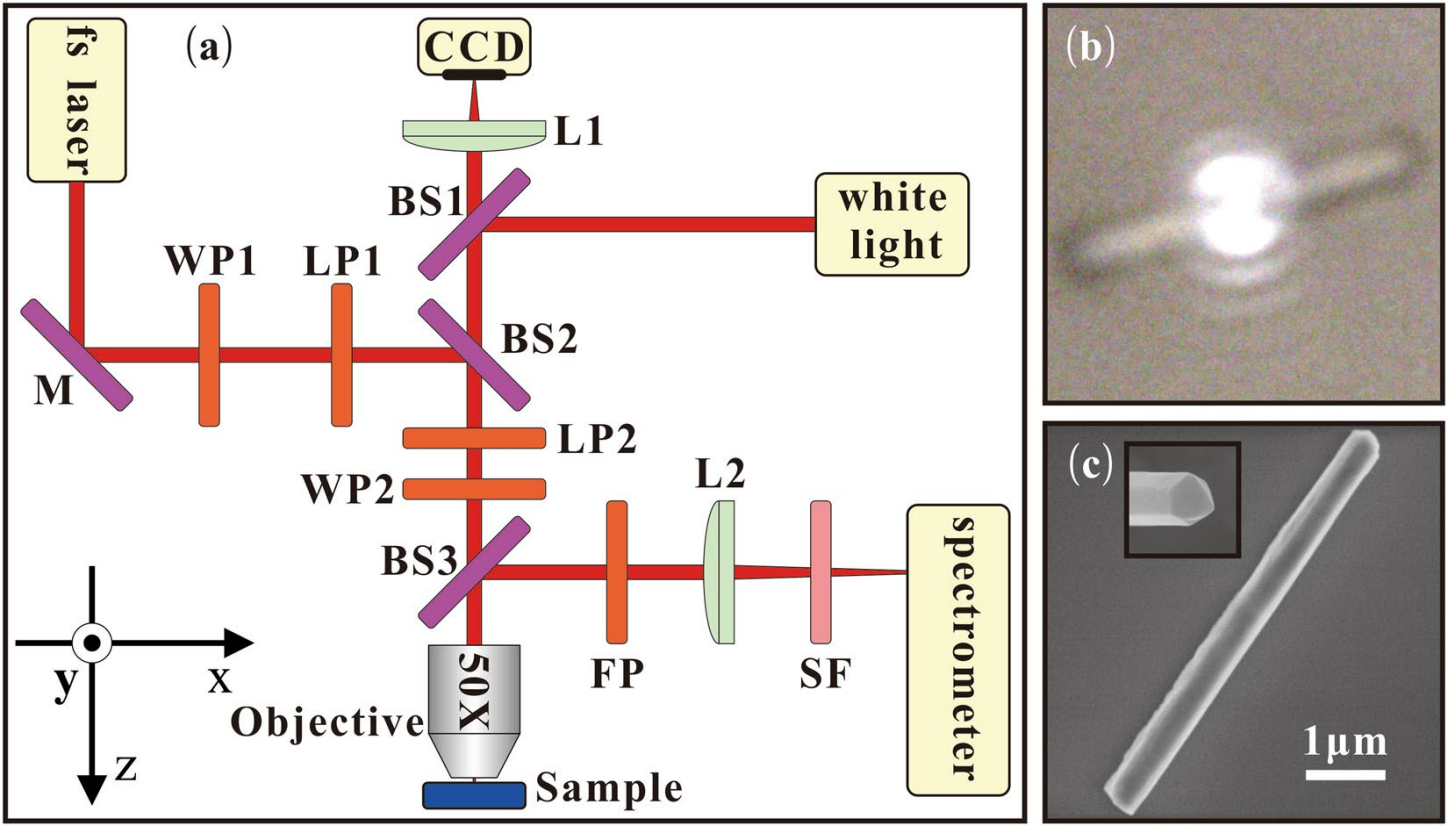
(**a**) Schematic diagram showing the experimental setup used to measure SHG from a single GaAs NW. M: mirror; WP1, WP2: half-wave plates; LP1, LP2, FP: polarizers; BS1, BS2, BS3: beam splitters; L1, L2: lenses; SF: short-pass filter. (**b**) CCD image of a GaAs NW with the pump laser spot focused on it. (**c**) SEM image of single GaAs NW on a SiO_2_ substrate, where the NW has a diameter of approximately 500 nm and length of approximately 5.55 *μ*m. The inset shows the hexagonal NW cross-section.

We present SHG from a single hexagonal GaAs NW that was measured using a homemade confocal microscope system. The experimental setup is illustrated schematically in Fig. 1(a). Here the fs laser, i.e. the fundamental wave (FW) laser, is generated using a mode-locked Ti:sapphire laser (MaiTai HP, Spectra Physics) with a pulse width of <100 fs, a tuning range of 690 to 1040 nm, and a repetition rate of 80 MHz. The experimental setup that was used to measure SHG from single GaAs NWs is described in greater detail in the Methods section.

The GaAs NW with $\bar{4}3m$ space group that was studied was synthesized on GaAs (100) substrates using the Veeco Mod Gen-II molecular beam epitaxy (MBE) system, with the vapour-liquid-solid (VLS) mechanism (further details of this process can be found in the Methods section). The NW was then dry-transferred to a Si/SiO_2_ substrate with a patterned gold grid that was used for spatial separation and identification. The SHG signal that was excited from the NW was subsequently imported into a charge-coupled device (CCD) or spectrometer. The occurrence of coupling effects between different GaAs NWs is excluded because of the focused laser spot size of ~1.5 *μ*m (Fig. 1(b)). Figure 1(c) shows a typical scanning electron microscope (SEM) image of a single GaAs NW with a diameter of approximately 500 nm and a length of approximately 5.55 *μ*m. The inset in Fig. 1(c) shows that the NW has a hexagonal cross-section as previously reported^35^.

**Figure 2:**
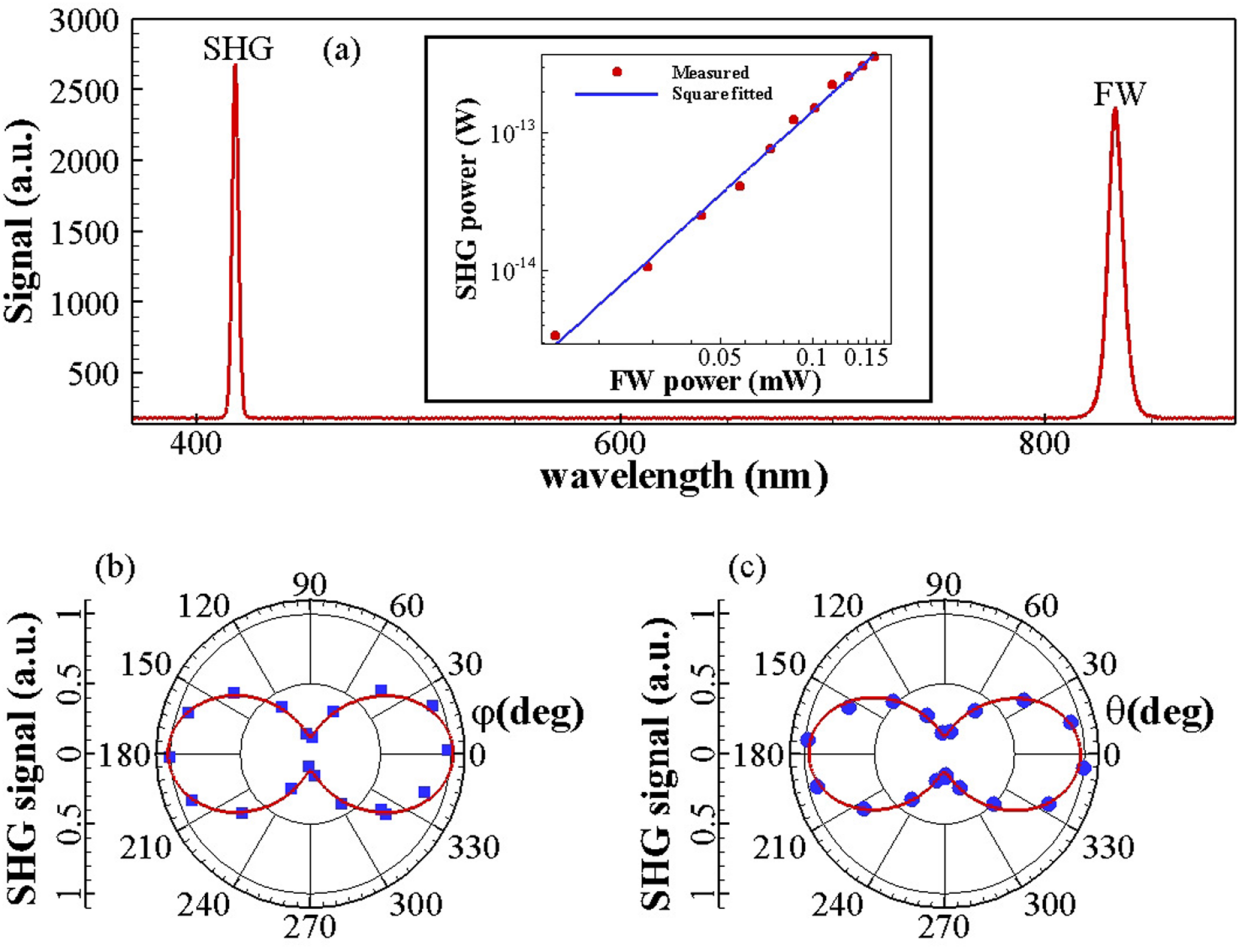
(**a**) Spectra of the excited SHG and the FW laser at a FW laser power of 87.12 *μ*W. The inset shows that the SHG signal has a square power dependence on the FW laser. (**b**) Polarimetric plot of the total SHG signal under parallel polarized excitation (TM). (**c**) Total SHG signal as a function of the FW polarization angle.

Micro-photoluminescence spectra were measured from an individual NW at room temperature under 80 MHz pulsed excitation. As shown in Fig. 2(a), the prominent peak at 417 nm, which represents the exactly frequency-doubling signal from the FW laser (834 nm), indicates that the signal is generated through a second-order nonlinear process. Additionally, no peaks were observed from defect-related photoluminescence emissions or two-photon excited fluorescence, which indicates the high crystalline quality of the GaAs NWs used^16^. The inset of Fig. 2(a) shows the signal intensity as a function of the power of the FW laser, where the quadratic dependency confirms that the signal can be ascribed to the SHG process in the single GaAs NW.

The polarization of the SHG signals was also studied. Figure 2(b) shows the measured SHG intensity as a function of signal polarization angle *φ* under a parallel polarized FW laser (transverse magnetic, TM) input. Here the polarization angle *φ* is defined as the angle of the electric field (E-field) direction of the SHG with respect to the long axis of the NW. The SHG intensity follows a cos^2^ *φ* shape where the peak is always polarized along the long axis of the NW. More detailed theoretical calculations are provided in the Supplementary Information section. The intensity characteristic thus allows the NW’s long axis to be identified, and we also investigated the SHG intensity as a function of the FW laser polarization angle *θ*, with results as shown in Fig. 2(c). In this case, the polarization angle *θ* is defined as the angle of the E-filed direction of the FW laser with respect to the long axis of the NW. The polarization direction is along the long axis of the NW (i.e., where *θ* = 0), which indicates that the anisotropy of the SHG can be attributed to the intrinsic permutation symmetry of the GaAs crystal lattice.

**Figure 3:**
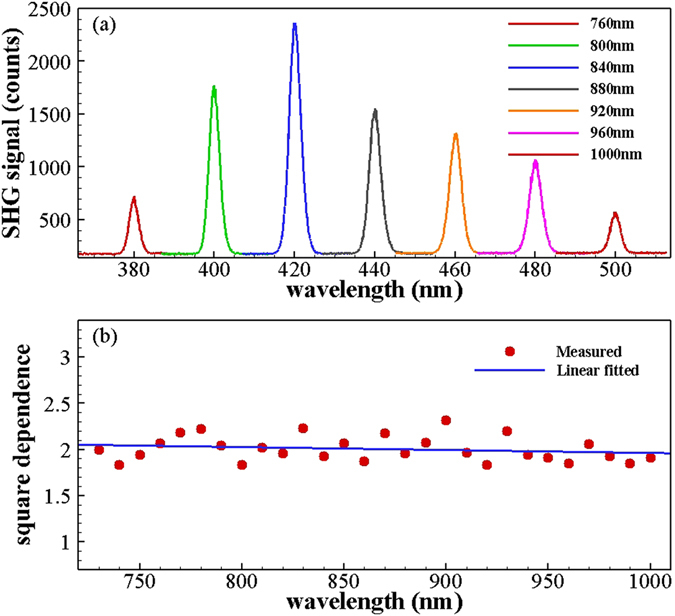
(**a**) SHG at a range of discrete FW wavelengths with constant FW power of 4.07 mW. (**b**) Square power dependence of the SHG signal on the FW laser demonstrated at each discrete FW wavelength from 730 nm to 1000 nm.

To illustrate the origin of the SHG in the nanowires, we then studied the SHG signals at different wavelengths under TM excitation. As shown in Fig. 3(a), SHG in the single GaAs NW was detected at a series of discrete FW wavelengths over the range from 760 to 1000 nm at a constant FW power of 4.07 mW. The longest and shortest wavelengths obtained were 500 nm and 365 nm, which were excited by 1000 nm and 730 nm fs laser inputs, respectively. The results indicate that the single GaAs NW can be excited to generate second harmonic light by the fs laser over a broad wavelength range. As shown in Fig. 3(b), the SHG signal has a quadratic power dependence on the FW laser over the FW wavelength range, which indicates that this is a second-order nonlinear process. Most importantly, this broadband SHG process demonstrates wavelength insensitivity, which can be attributed to surface SHG that is generated within a nanoscale region without signal propagation and therefore does not require phase matching (PM) conditions^20^.

**Figure 4:**
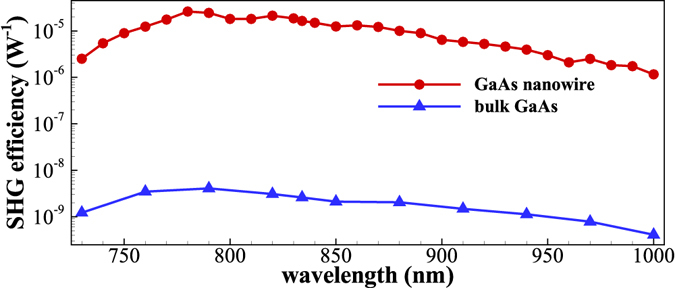
Conversion efficiency of SHG signals over a wide FW wavelength range.

To confirm these results, we also investigated the conversion efficiency *η*_2*ω*_ of the SHG signal, which is defined in this work as ${\eta }_{2\omega }={P}_{2\omega }/{P}_{\omega }^{2}$, where *P*_2*ω*_ and *P*_*ω*_ are the SHG signal and FW laser powers, respectively. The method used to measure the conversion efficiency can be found in the Methods section. It was previously established that broadband SHG often comes at the cost of reduced conversion efficiency^36^. High-efficiency SHG depends critically on the PM conditions. However, the PM requirement limits significantly the bandwidth of the SHG processes^37^. Thus, the high-efficiency broadband SHG has never been really obtained to the best of our knowledge. Herein we demonstrated that the broadband SHG observed in the single hexagonal GaAs NW was simply a high-efficiency SHG process. We found that the SHG conversion efficiency of the single GaAs NW maintained a high value over a wide FW wavelength range. Figure 4 shows typical experimental results, where the SHG conversion efficiency of the single GaAs NW is three orders of magnitude higher than that at the surface of bulk GaAs over the wavelength range from 730 nm to 1000 nm, and can reach the order of ~10^−5^ W^−1^. The high SHG conversion efficiency of the single GaAs NW probably stems from its high surface-to-volume ratio. This ratio breaks the crystal symmetry and induces the dipoles to oscillate with the E-field of the FW laser. The second-order nonlinearity coefficient of the NW is therefore enhanced. Additionally, the NW structure offers confinement and surface enhancement effects to the laser E-field. The frequency conversion in the single GaAs NW is therefore more efficient than that which occurs at the surface of bulk GaAs^34^.

**Figure 5:**
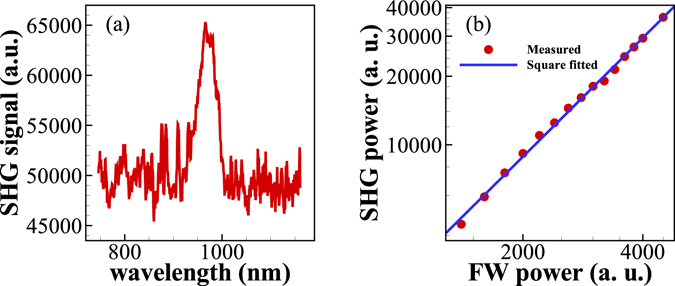
(**a**) SHG signal excited using 1960 nm FW laser. (**b**) Square power dependence of the SHG signal on the FW laser input.

As an additional trial, a fs-scale optical parametric amplifier (OPerA Solo, Coherent) with a pulse width of <100 fs, a tuning range of 190 to 20000 nm, and a repetition rate of 1 kHz was used to excite the single hexagonal GaAs NW. The experimental setup was almost the same as that shown in Fig. 1(a). The main difference was that the intensity of the FW laser was adjusted not by the half-wave plate (WP1) and the linear polarizer (LP1) but by a neutral density filter because of a lack of available half-wave plates and linear polarizers that operate in this wavelength range. The half-wave plate (WP2), linear polarizer (LP2), and film polarizer (FP) were removed from the optical path because we do not measure a polarimetric plot of the SHG signal in this wavelength range. The short-pass filter (SF) was replaced with a model 84–655 filter from Edmund Optics to filter out the FW laser light in this wavelength range. The CCD camera used to observe the NW was replaced with a Micronviewer from Electrophysics. The spectrometer was also replaced with a model USB2000+ from Ocean Optics to measure the micro-photoluminescence spectra from the NW. Because of the restrictions of these devices, the longest SHG wavelength that was obtained was 980 nm, which was excited by a 1960 nm FW laser input, as shown in Fig. 5(a). Figure 5(b) shows that the SHG signal has a square power dependence on the FW laser input, which indicates that the SHG is indeed a second-order nonlinear process.

In summary, we have investigated SHG in a single hexagonal GaAs NW in detail. We found that excitation of this NW can generate second harmonic light that has not been simultaneously accompanied by defect-related photoluminescence emission or two-photon excited fluorescence to date. Additionally, the generated SHG is highly coherent and has very good polarization properties. We observed SHG over a broad wavelength range from 700 nm to 1960 nm, which is the widest wavelength range to be demonstrated in NWs to the best of our knowledge. The SHG conversion efficiency is also very high and can reach the order of ~10^−5^ W^−1^, which is ~10^3^ times higher than that obtained from the surface of bulk GaAs. This high-efficiency broadband SHG procedure based on a single NW may find applications in imaging, bio-sensing and on-chip all-optical signal and processing.

## Methods

### Optical measurements

We performed a series of experiments to measure SHG in a single GaAs NW using a homemade confocal microscope system. The experimental setup is illustrated schematically in Fig. 1(a). The fs laser is generated using a mode-locked Ti:sapphire laser (MaiTai HP, Spectra Physics) with a pulse width of <100 fs, a tuning range of 690 to 1040 nm, and a repetition rate of 80 MHz. The FW laser intensity was adjusted by rotating the half-wave plate (WP1, AHWP10M-980, Thorlabs) while the linear polarizer (LP1, LPVIS100-MP2, Thorlabs) remained fixed, and the laser polarization was controlled by rotating a second half-wave plate (WP2). The linear polarizer (LP2) that was placed in front of the half-wave plate is used to refine the polarization. The FW laser was focused on individually selected NWs using a microscope objective (50X, numerical aperture of 0.8, Olympus). The excited signal that was emitted by the NW was collected using the same objective and was reflected using a beam splitter (BS3) through a 150 mm-focal-length lens (L2) to a spectrometer/monochromator (Acton SP2750, Princeton) that was equipped with a liquid-nitrogen-cooled CCD (Spec-10:100BR, Princeton). A short-pass filter (SF, FF01, Semrock, S810, Asahi Spectra, or FESH0900, Thorlabs) was placed in front of the spectrometer to filter out the FW laser light. The polarimetric plot of the total SHG signal under parallel polarized excitation (TM) was measured by simply rotating the film polarizer (FP, LPVISE100-A, Thorlabs). The CCD camera (Infinity3, Lumenera) was used to acquire images of the sample via the lens (L1) under white light illumination, to enure that the FW laser was normally incident on the NW.

### Nanowire growth

The GaAs NWs under study were synthesized on GaAs (100) substrates using the Veeco Mod Gen-II MBE system and the VLS mechanism. The substrates were first coated by sputtering of a 15-nm-thick silicon dioxide layer and were then dipped for 2 s in a 10% HF aqueous solution. Prior to growth, the substrates were degassed at 700 °C for 10 min and growth was then initiated using 1 nm Ga droplets as a catalyst at 620 °C in the absence of arsenic overpressure. The GaAs core was grown at 620 °C for 30 min at an As_2_/Ga flux ratio of 12.5; a 10 min interruption was then introduced under a high arsenic atmosphere to crystallize the gallium droplets on the tip and induce lateral growth of the nanowire. The deposition rate (equivalent to the planar growth rate on a GaAs (100) substrate) of these GaAs NWs was set at 0.7 *μ*m/h.

### SHG signal conversion efficiency

To obtain accurate measurements of the SHG conversion efficiency *η*_2*ω*_, we first detected the power *P*_*ω*_ of the FW laser using a power meter. Second, we recorded number of photons N of the SHG signal using the spectrometer and calculated the power *P*_2*ω*_ of the SHG signal using the formula ${P}_{2\omega }=(Nhc/\lambda )/t$, where *h* is Planck’s constant, *c* is the speed of light and *λ* is the wavelength of the SHG signal. We then calculated the SHG conversion efficiency *η*_2*ω*_, based on the definition ${\eta }_{2\omega }={P}_{2\omega }/{P}_{\omega }^{2}$ for the SHG conversion efficiency^26^.

## Electronic supplementary material

High-efficiency broadband second harmonic generation in single hexagonal GaAs nanowire
