# Supplementary material for: High-efficiency broadband second harmonic generation in single hexagonal GaAs nanowire
Source: Sci Rep. 2017 May 19;7:2166. doi: 10.1038/s41598-017-02199-w (PMC5438389; doi:10.1038/s41598-017-02199-w)
Supplement: Supplementary file 1 — High-efficiency broadband second harmonic generation in single hexagonal GaAs nanowire [file 41598_2017_2199_MOESM1_ESM.pdf]

# High-efficiency broadband second harmonic generation in single hexagonal GaAs nanowire

**Jing Wang[1], Yu-Ming Wei[1], Shun-Fa Liu[2], Ying Yu[2,*], Juntao Li[1], Zhang-Kai Zhou[1], Zhi-Chuan Niu[3], Si-Yuan Yu[2,4], and Xue-Hua Wang[1,*]**

[1]State Key Laboratory of Optoelectronic Materials and Technologies, School of Physics, Sun Yat-Sen University, Guangzhou 510275, China
[2]State Key Laboratory of Optoelectronic Materials and Technologies, School of Electronics and Information Technology, Sun Yat-Sen University, Guangzhou 510275, China
[3]State Key Laboratory of Superlattices and Microstructures Institute of Semiconductors, Chinese Academy of Sciences, Beijing, 100083, China
[4]Photonics Group, Merchant Venturers School of Engineering, University of Bristol, Bristol BS8 1UB, UK
[*]Correspondence and requests for materials should be addressed to Y.Y.(yuying26@mail.sysu.edu.cn) or X.H.W.(wangxueh@mail.sysu.edu.cn)

## The polarization of the SHG signals

The electric field of second harmonic generation (SHG) signal, $\overrightarrow{E_{2\omega}}$ ($E_{2\omega,cx}$, $E_{2\omega,cy}$, $E_{2\omega,cz}$), can be described by the matrix,

$$\begin{pmatrix} E_{2\omega,cx} \\ E_{2\omega,cy} \\ E_{2\omega,cz} \end{pmatrix} = \begin{pmatrix} d_{11} & d_{12} & d_{13} & d_{14} & d_{15} & d_{16} \\ d_{21} & d_{22} & d_{23} & d_{24} & d_{25} & d_{26} \\ d_{31} & d_{32} & d_{33} & d_{34} & d_{35} & d_{36} \end{pmatrix} \begin{pmatrix} E^2_{\omega,cx} \\ E^2_{\omega,cy} \\ E^2_{\omega,cz} \\ 2E_{\omega,cy}E_{\omega,cz} \\ 2E_{\omega,cz}E_{\omega,cx} \\ 2E_{\omega,cx}E_{\omega,cy} \end{pmatrix}, \tag{1}$$

where $d_{ij}$ represents second-order nonlinear tensor of the nanowire, $E_{\omega,cx}$, $E_{\omega,cy}$, and $E_{\omega,cz}$ denote the components of the electric field $\overrightarrow{E_{\omega}}$ of the fundamental wave (FW) laser, and the subscript $\omega$ and $2\omega$ stand for the frequency of FW and SHG signal respectively.

As GaAs nanowire belongs to the point group $4\bar{3}$m, its second-order nonlinear coefficient is written as,

$$d_{ij} = \begin{pmatrix} 0 & 0 & 0 & d_{14} & 0 & 0 \\ 0 & 0 & 0 & 0 & d_{14} & 0 \\ 0 & 0 & 0 & 0 & 0 & d_{14} \end{pmatrix}. \tag{2}$$

Substituting Eq.(2) into Eq.(1) and obtaining,

$$\begin{pmatrix} E_{2\omega,cx} \\ E_{2\omega,cy} \\ E_{2\omega,cz} \end{pmatrix} = 2d_{14} \begin{pmatrix} E_{\omega,cy}E_{\omega,cz} \\ E_{\omega,cz}E_{\omega,cx} \\ E_{\omega,cx}E_{\omega,cy} \end{pmatrix}. \tag{3}$$

Considering the coordinate systems: the lab coordinate system ($\vec{e}_x$, $\vec{e}_y$, $\vec{e}_z$), where the nanowire is on the z-y plane. Then the components of SHG which is parallel ($\overrightarrow{E(0^\circ,\theta)}$) or perpendicular ($\overrightarrow{E(90^\circ,\theta)}$) to the nanowire is written as,

$$\overrightarrow{E(0^\circ,\theta)} = (\overrightarrow{E_{2\omega}} \cdot \vec{e}_z)\vec{e}_z \tag{4}$$

$$\overrightarrow{E(90^\circ,\theta)} = (\overrightarrow{E_{2\omega}} \cdot \vec{e}_y)\vec{e}_y \tag{5}$$

In addition, the SHG intensity, whose polarization is at an angle of $\varphi$ with respect to $\vec{e}_z$ in the y-z plane, is given by the expression,

$$I_{2\omega}(\varphi,\theta) = |\overrightarrow{E_{2\omega,p}}\cos\varphi + \overrightarrow{E_{2\omega,s}}\sin\varphi|^2 \tag{6}$$

In the case of $\theta = 0^\circ$, $\overrightarrow{E_{2\omega,p}} = 2d_{14}E^2_{\omega}/\sqrt{3}$, $E_{2\omega,s} = 0$, Thus,

$$I_{2\omega}(\varphi,0^\circ) = |\overrightarrow{E_{2\omega,p}}(0^\circ)\cos\varphi + \overrightarrow{E_{2\omega,s}}(0^\circ)\sin\varphi|^2 = \frac{4}{3}d^2_{14}E^4_{\omega}\cos^2\varphi \tag{7}$$

## Comparisons of SHG reported for different nanostructures

| Nanostructures | GaAs nanowire | CdS nanowire[1] | Au nanocup[2] |
|---|---|---|---|
| Diameter D ($\mu$m) Length L ($\mu$m) | D=0.5, L=5.55 | D=0.23, L=2 | D=0.12 |
| Nonlinear coefficient $\|d_{ij}\|$ (pmV$^{-1}$) | 170 or 370 | 36, 19 or 78 | 3.2 |
| Conversion efficiency $\eta_{2\omega}$ (W$^{-1}$) | $\sim 10^{-5}$ | $9.2 \times 10^{-9}$ | $< 10^{-9}$ |

**Table 1.** Comparisons of SHG signal conversion efficiency for different nanostructures.

As is known, the conversion efficiency of SHG signal depends on the devices' nonlinear coefficients ($d_{ij}$) and sizes (Diameter D or Length L).[1] As shown in TABLE I, the conversion efficiency of SHG signal in GaAs nanowire is higher than that in CdS nanowire and Au nanocup because of its larger nonlinear coefficients and surface-to-volume ratio.

## References


1. Ren, M. L., Liu, W. J., Aspetti, C. O., Sun, L. X., & Agarwal, R. Enhanced second-harmonic generation from metal-integrated semiconductor nanowires via highly confined whispering gallery modes. Nat. Commun. **5,** 5432 (2014).
2. Zhang, Y., Grady, N. K., Ayala-Orozco, C. & Halas, N. J. Three-Dimensional Nanostructures as Highly Efficient Generators of Second Harmonic Light. Nano Lett. **11,** 5519-5523 (2011).
